# Post mastectomy linac IMRT irradiation of chest wall and regional nodes: dosimetry data and acute toxicities

**DOI:** 10.1186/1748-717X-8-81

**Published:** 2013-04-08

**Authors:** Jinli Ma, Jiongyan Li, Jiang Xie, Jian Chen, Chuanying Zhu, Gang Cai, Zhen Zhang, Xiaomao Guo, Jiayi Chen

**Affiliations:** 1 Department of Radiation Oncology, Fudan University Shanghai Cancer Center, Cancer Institute of Fudan University, Shanghai, 200032, China

**Keywords:** Breast cancer, Modified radical mastectomy, Intensity modulation, Dosimetry, Acute toxicity

## Abstract

**Background:**

Conventional post-mastectomy radiation therapy is delivered with tangential fields for chest wall and separate fields for regional nodes. Although chest wall and regional nodes delineation has been discussed with RTOG contouring atlas, CT-based planning to treat chest wall and regional nodes as a whole target has not been widely accepted. We herein discuss the dosimetric characteristics of a linac IMRT technique for treating chest wall and regional nodes as a whole PTV after modified radical mastectomy, and observe acute toxicities following irradiation.

**Methods:**

Patients indicated for PMRT were eligible. Chest wall and supra/infraclavicular region +/−internal mammary nodes were contoured as a whole PTV on planning CT. A simplified linac IMRT plan was designed using either integrated full beams or two segments of half beams split at caudal edge of clavicle head. DVHs were used to evaluate plans. The acute toxicities were followed up regularly.

**Results:**

Totally, 85 patients were enrolled. Of these, 45 had left-sided lesions, and 35 received IMN irradiation. Planning designs yielded 55 integrated and 30 segmented plans, with median number of beams of 8 (6–12). The integrated and segmented plans had similar conformity (1.41±0.14 vs. 1.47±0.15, *p*=0.053) and homogeneity indexes (0.13±0.01 vs. 0.14±0.02, *p*=0.069). The percent volume of PTV receiving >110% prescription dose was <5%. As compared to segmented plans, integrated plans typically increased V_5_ of ipsilateral lung (*p*=0.005), and heart (*p*=0.001) in patients with left-sided lesions. Similarly, integrated plans had higher spinal cord D_max_ (*p*=0.009), ipsilateral humeral head (*p*<0.001), and contralateral lung D_mean_ (*p*=0.019). During follow-up, 36 (42%) were identified to have ≥ grade 2 radiation dermatitis (RD). Of these, 35 developed moist desquamation. The median time to onset of moist desquamation was 6 (4–7) weeks from start of RT. The sites of moist desquamation were most frequently occurred in anterior axillary fold (32/35), and secondly chest wall (12/35). The difference in occurrence of ≥ grade 2 RD between integrated and segmented plans was statistically insignificant (*X*^2^=0.35, *p*=0.55). Only 2 were found to have grade 2 radiation pneumonitis.

**Conclusions:**

The linac IMRT technique applied in PMRT with chest wall and regional nodes as a whole PTV was dosimetrically feasible, and the treatment was proved to be well-tolerated by most patients.

## Background

Although the utilization of breast conserving surgery (BCS) for early-stage disease has increased rapidly in last decade in mainland China, modified radical mastectomy (MRM) remains the most-accepted surgical modality in operable breast cancer [1]. Three randomized clinical trials have shown that a disease-free and overall survival advantage is conferred by the addition of chest wall and regional lymph node irradiation in women with positive axillary lymph nodes after MRM [2-4].

Conventional post-mastectomy radiation therapy (PMRT) is often delivered with traditional field borders for chest wall and regional nodes. Although chest wall and regional nodes delineation techniques have been discussed with available contouring guidelines, computed tomography (CT)-based planning to treat chest wall and nodal regions as a whole PTV has not yet been adopted into routine practice. We herein discuss the dosimetric characteristics of an inverse-planned intensity-modulated radiation therapy (IMRT) technique for total local-regional irradiation after MRM, and observe the acute toxicities following irradiation.

## Methods

### Patient eligibility

Eligibility criteria included: (1) age ≥ 18 years with operable breast cancer involving axillary lymph nodes, but without evidence of distant metastasis (negative results on chest CT scans, abdomen and pelvis US); (2) resection of all gross disease by MRM with level I to II axillary dissection; (3) negative surgical margins; (4) Eastern Cooperative Oncology Group performance score of 0–1; (5) completion of adjuvant chemotherapy; and (6) no previous thoracic RT. Patients with serious comorbid diseases, such as chronic obstructive pulmonary disease, connective tissue disease, postoperative wound infections, and delayed wound healing, etc., that would have negatively affected their tolerance to radiation-induced skin or lung toxicity were not eligible. Patients with synchronous bilateral breast cancers were eligible. All patients provided written informed consent.

### Study design

There were two parts to this study. The first part was to discuss dosimetric characteristics of a PMRT method, which would treat chest wall and regional nodes as a whole PTV with linac IMRT technique (dosimetric study). And the second part was to evaluate acute toxicities following PMRT with total local-regional IMRT (clinical study).

### Dosimetric study

#### Images acquisition

Before simulation, the patient was placed supine on a commercially available breast tilt board (Med-Tech 350) to make sternum parallel to the table, with both arms fully abducted (90 degrees or greater) and externally rotated, and head position secured. A planning CT scan at 5-mm intervals from mid-neck to diaphragm with no contrast enhancement was obtained for each patient using an AcQsim CT simulator (Philips Medical Systems). At simulation, the mastectomy scar was routinely wired with radiopaque markers.

#### Contours definition

The clinical target volumes (CTV) were defined to consist of ipsilateral chest wall, mastectomy scar, and supra/infraclavicular region for each patient. Treatment of internal mammary nodes (IMN) was strongly considered when primary tumor was located in central or medial part of the breast. Each CTV was delineated according to the breast cancer atlas for radiation therapy planning consensus definitions of the Radiation Therapy Oncology Group (RTOG) (available at: http://www.rtog.org/CoreLab/ContouringAtlases/BreastCancerAtlas.aspx). The borders of CTV for chest wall and regional nodes were described in Table 1. The chest wall CTV was expanded 1 cm to become chest wall PTV, except that anterior, posterior and cranial borders were unchanged. This modification was made mainly to account for build-up region or spare underling normal lung from high dose radiation [5,6]. If IMN irradiation not indicated, medial border of chest wall PTV was usually at medial edge of sternal-rib junction (with reference to medial border of contralateral breast). The anatomically based guidelines established by Dijkema et al. [7] were also referenced when regional node CTVs were contoured. Generally, expansions of 5 mm to CTV for supra/infraclavicular nodes and 7 mm to CTV for IMN were made to form the PTV for supra/infraclavicular nodes and IMN, respectively. If IMN included, PTV for IMN would be smoothly integrated into PTV for chest wall. The PTV for supra/infraclavicular nodes would match the PTV for chest wall, and IMN if indicated, at the clavicle head inferiorly. As a result, a whole PTV including both chest wall and regional nodes formed.

**Table 1:** Anatomic borders of CTV for chest wall and regional nodes

**Structures**	**Cranial**	**Caudal**	**Anterior**	**Posterior**	**Lateral**	**Medial**
Chest wall	Caudal border of the clavicle head	Contralateral inframammary fold	2 mm below the skin surface	Rib-pleural interface	Mid-axillary line	Sternal-rib junction
Supra-clavicular	Caudal to the cricoid cartilage	Junction of brachioecph.- axillary veins/caudal edge clavicle head	Posterior edge of SCM muscle	Anterior aspect of the scalene muscle	Cranial: lateral edge of SCM muscle	Excludes thyroid and trachea
					Caudal: junction 1st rib-clavicle	
Infra-clavicular	Pectoralis minor muscle insert on coracoid	Axillary vessels cross medial edge of pectoralis minor muscle	Posterior surface of pectoralis major muscle	Ribs and intercostal muscles	Medial border of pectoralis minor muscle	Thoracic inlet
Internal mammary	Superior aspect of the medial 1st rib.	Cranial aspect of the 4th rib	Encompass the internal mammary vessels	Encompass the internal mammary vessels	Encompass the internal mammary vessels	Encompass the internal mammary vessels

Abbreviations: *SCM*= sternocleidomastoid.

The organs at risk (OARs) surrounding the targets, including bilateral lungs, heart, contralateral breast, ipsilateral humeral head, spinal cord, and esophagus, were contoured as well. The heart was defined as from its apex to the junction of great vessels with myocardium, and esophagus was contoured from cricoid cartilage to cardia. In addition, the healthy tissue was defined as the patient’s volume covered by the CT scan minus the envelope of the PTV to account for the spillage of prescription dose.

#### Plan optimization

**Figure 1:**
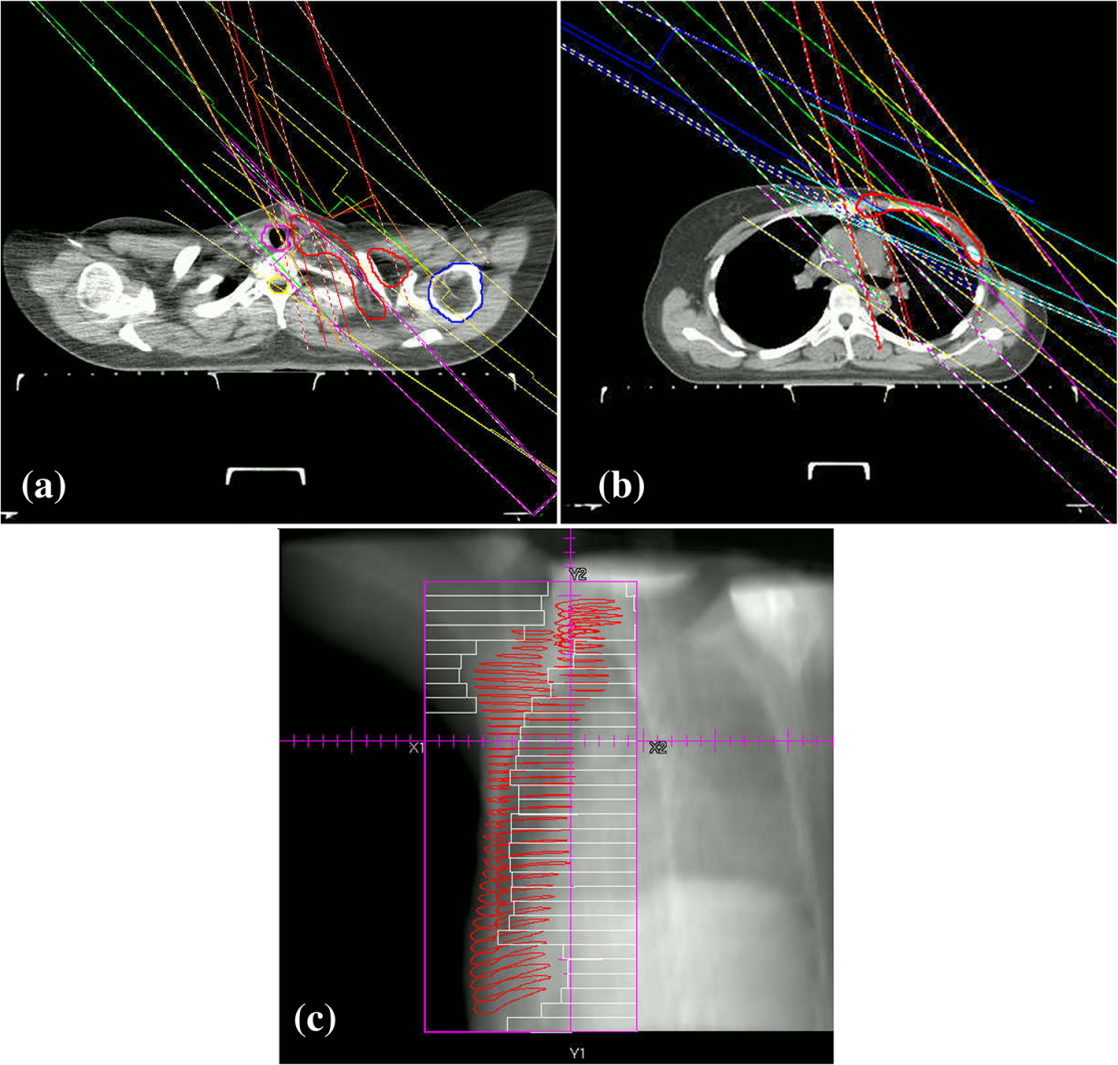
A 7-beam integrated plan designed for a patient with left breast cancer: (a) beam angles (115, 125,135, 300, 315, 330, and 345 degrees) for supra/infraclavicular region; (b) identical beam angles for chest wall and IMN; (c): lateral beam’s-eye-view (BEV) of the whole PTV with multi-leaf collimator (MLC)-defined port displayed.

**Figure 2:**
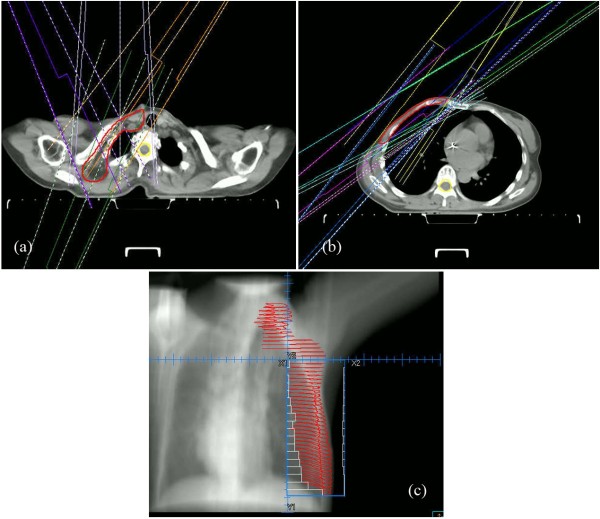
A 10-beam segmented plan designed for a patient with right breast cancer: (a) 4 beams (0, 35, 215, and 330 degrees) for supra/infraclavicular region; (b) six beams (35, 45, 55, 220, 235, and 245 degrees) for chest wall; (c): lateral BEV of the PTV with MLC-defined port displayed.

For each patient, a multi-beam simplified IMRT plan was generated using Pinnacle treatment planning software (version 8.0). The IMRT plans were designed using either integrated full beams or two segments of half beams split at caudal edge of clavicle head, the latter technique require that the length of chest wall PTV should not exceed 19 cm so as to be fully covered by the half beams at caudal side. Although the selection of beam angles was at discretion of the responsible dosimetrist, basically, an integrated plan employs full beam to cover the whole PTV, whereas a segmented plan uses different beam angles in the cranial half beams to cover the upper part of PTV (supra/infraclavicular region), and caudal half beams to cover the lower part (chest wall±IMN), respectively. The angles sectors covered by multiple beams are shown in Figures 1 and 2 for representative integrated and segmented plans, respectively. All plans were optimized to cover the whole PTVs and spare surrounding normal tissues as much as possible. To ensure a sufficient skin dose, a daily 3-mm bolus was placed on chest wall of each patient. The optimization process started with dose-volume constraints as follows: 90% of PTV to receive 50Gy in 25 fractions; ipsilateral mean lung dose ≤20Gy, and ≤30% of the ipsilateral lung to receive ≤20Gy; ≤5% of the heart to receive ≤30Gy, mean heart dose ≤8Gy for left-sided lesions, and ≤10Gy if IMN was included; spinal cord maximum dose ≤45Gy; contralateral breast mean dose ≤1.5Gy. Priority was high for the PTV, heart and lung constraints relative to other structures. Optimization proceeded with these settings until no further improvement was seen. Priority was then increased for other structures until a balance was reached between PTV coverage and normal tissues sparing. During optimization, a simplified IMRT plan was defined to have ≤5 segments/beam, ≥ 10 cm^2^/segment, and ≥10 MU/segment [8]. After optimization, a final dose calculation using the collapsed cone convolution superposition (CCCS) algorithm was performed. Dose grid size used for calculations was 0.2 cm by 0.2 cm by 0.2 cm. Once PTV and normal tissues dose constraints were met, the dosimetrist would expand the anterior border of chest wall field 1.5-2 cm beyond the skin surface to ensure chest wall coverage.

#### Plan evaluation

For dosimetric analysis, the following indices extracted from dose-volume histograms (DVHs) were used: 1) D_near-max_, D_near-min_, and D_mean_ for PTV: D_near-max_ is defined to be the dose to the 2% of the PTV (D_2%_), D_near-min_ is the dose to the 98% of the PTV (D_98%_), and _Dmean_ is the mean dose to the PTV; 2) V_95%_, V_107%_ and V_110%_ for PTV: percent volume receiving greater than 95% to 110% of prescribed dose; 3) dose homogeneity index (HI) and conformity index (CI) for PTV: HI and CI were calculated according to definition proposed by the International Commission on Radiation Units and Measurements (ICRU) [9] and expressed as follows: $\text{HI}=\frac{D2\%-D98\%}{D50\%}\times 100\%$ and $\text{CI}=\frac{\text{Precription}\;\text{Isodose}\;\text{Volume}}{\text{Target}\;\text{Volume}}$, lower HI and CI correlate with a more homogeneous target dose and better conformity, respectively; 4) ipsilateral lung V_5_, V_20_, and D_mean_; contralateral lung V_5_ and D_mean_; heart V_5_, V_10_, V_20_, V_30_, D_mean_; spinal cord D_max_; ipsilateral humeral head D_mean_; esophagus D_near-max_ and D_mean_; contralateral breast V_5_ and D_mean_; 5) external volume index (EI) for healthy tissue [10]: EI is defined as V_healthy tissue_/V_PTV_ where V_healthy tissue_ is the volume of healthy tissue receiving more than 50Gy and V_PTV_ is the volume of the total PTV.

### Clinical study

All treatments were delivered with 6-MV photon using an Electa linear accelerator. To ensure accurate delivery of each IMRT plan, orthogonal megavoltage electronic portal images were captured once before the first treatment and per week thereafter, and compared with reference digitally reconstructed radiographs (DRRs) to verify patient position. Each patient was regularly followed up by the treating physician once a week during radiotherapy and after irradiation. Radiation dermatitis (RD) was checked up to 1 month after the completion of treatment. The occurring time and site of most serious radiation dermatitis were recorded for each patient. Radiation pneumonitis (RP) was assessed within 6 months. All acute side effects were graded according to the common terminology of criteria for adverse effects (CTCAE v4.0) issued by the National Cancer Institutes.

### Statistical analysis

The independent two-sample t-test was used to compare the dosimetric parameters between plans. Chi-square test was used to see the statistical significance of the difference in the occurrence of ≥grade 2 RD between integrated and segmented plans. *p* values of ≤ 0.05 were considered statistically significant.

## Results

Between June 2009 and Oct 2010, 87 patients were randomly enrolled onto the study and 85 patients (median age of 50 (28–69)) met eligibility for analysis. Of these, there were 45 cases with left-sided breast cancer, and 35 received IMN irradiation, including 19 left-sided lesions. All patients had IMRT plans approved by treating physicians.

### Dosimetry data

#### Delivery parameters

The median number of beams applied was 8 (range 6–12) for all patients. Of their plans, 55 were integrated, and 30 were segmented. The median number of beams was 7 (range 6–10) for integrated plans and 10 (range 8–12) for segmented plans which included 4 (range 3–6) beams for upper segments and 6 (range 4–7) beams for lower segments. The average of total MUs per fraction was 829.7±197.2. The number of MUs was significantly lower for integrated plans compared with segmented plans (728.9±111.3 vs. 1014.5±187.9, *p*<0.001).

#### Target coverage and homogeneity

Table 2 summarized the results of PTV dosimetry. In comparison with integrated plans, segmented plans had higher D_near-max_, D_mean_, V_107%_, and V_110%_; however, their dose conformity and homogeneity were similar. In addition, the V_110%_ was generally <5%, indicating that a very small volume of PTV received >110% of prescription dose. The inclusion of IMN in PTV didn’t compromise the target coverage and dose homogeneity.

**Table 2:** Summary of DVH-based analysis for the PTV

**Parameters**	**Plans**	**Value (Mean**±**SD)**	***P*-value**
D_near-max_ (Gy)	All	54.76±0.97	
	Integrated	54.57±0.88	***0.047***
	Segmented	55.01±1.05	
D_near-min_ (Gy)	All	47.53±0.63	
	Integrated	47.56±0.70	0.622
	Segmented	47.48±0.47	
D_mean_ (Gy)	All	51.76±0.41	
	Integrated	51.68±0.35	***0.011***
	Segmented	51.94±0.47	
V_95%_	All	98%±1%	
	Integrated	98%±1%	0.193
	Segmented	98%±1%	
V_107%_	All	9%±6%	
	Integrated	7%±5%	***0.003***
	Segmented	11%±6%	
V_110%_	All	2%±1%	
	Integrated	2%±2%	***0.023***
	Segmented	3%±2%	
HI	All	0.13±0.02	
	Integrated	0.13±0.01	0.069
	Segmented	0.14±0.02	
CI	All	1.43±0.15	
	Integrated	1.41±0.14	0.053
	Segmented	1.47±0.15	

Abbreviations: *PTV*: planning target volume; *D*_*near-max*_: maximum dose; *D*_*near-min*_: minimum dose; *Dmean*: mean dose; *CI*: conformity index; *HI*: homogeneity index; *Vx%*: percent volume of PTV receiving x% of prescription dose; *IMN*: internal mammary nodes.

#### OARs and healthy tissue

Table 3 listed the dose-volume statistics of OARs and healthy tissue. All IMRT plans were clinically acceptable regarding the dose-volume of lung, heart, spinal cord, and contralateral breast irradiated. As compared to segmented plans, integrated plans typically increased V_5_ of ipsilateral lung (*p*=0.005), and heart (*p*=0.001) in patients with left-sided lesions. Also, integrated plans had higher spinal cord D_max_ (*p*=0.009), D_mean_ of ipsilateral humeral head (*p*<0.001), and D_mean_ of contralateral lung (*p*=0.019). Doses to other OARs, such as esophagus D_near-max_ and contralateral breast D_mean_, were higher in integrated plans, although the differences did not reach statistical significance. In addition, compared to integrated plans, segmented plans reduced EI for healthy tissue.

**Table 3:** Summary of DVH-based analysis for OARs and healthy tissue

**Organ/Structure**	**Parameters**	**Plans**	**Value (Mean**±**SD)**	***P*-value**
Ipsilateral lung	V_5_	All	65%±8%	
		Integrated	67%±9%	***0.005***
		Segmented	61%±7%	
	V_20_	All	28%±2%	
		Integrated	28%±3%	0.980
		Segmented	28%±2%	
	D_mean_ (Gy)	All	15.06±1.66	
		Integrated	15.11±1.54	0.658
		Segmented	14.93±1.99	
Contralateral lung	V_5_	All	12%±11%	
		Integrated	14%±12%	0.157
		Segmented	9%±8%	
	D_mean_ (Gy)	All	2.26±1.32	
		Integrated	2.58±1.43	***0.019***
		Segmented	1.66±0.83	
Heart	V_5_	Left-sided lesions	56%±15%	
		Integrated	60%±13%	***0.001***
		Segmented	45%±13%	
	V_10_	Left-sided lesions	30%±9%	
		Integrated	31%±10%	0.076
		Segmented	26%±7%	
	V_20_	Left-sided lesions	13%±6%	
		Integrated	14%±7%	0.77
		Segmented	13%±4%	
	V_30_	Left-sided lesions	7%±3%	
		Integrated	8%±4%	0.21
		Segmented	6%±3%	
	D_mean_ (Gy)	Left-sided lesions	8.69±1.47	
		Integrated	8.76±1.61	0.65
		Segmented	8.54±1.14	
Spinal cord	D_max_ (Gy)	All	36.10±5.87	
		Integrated	37.24±4.55	***0.009***
		Segmented	33.29±7.97	
Ipsilateral humeral head	D_mean_ (Gy)	All	23.98±9.25	
		Integrated	28.22±5.60	***<0.001***
		Segmented	12.31±6.99	
Esophagus	D_near-max_ (Gy)	All	41.56±7.79	
		Integrated	41.84±9.15	0.85
		Segmented	41.29±6.47	
	D_mean_ (Gy)	All	11.14±3.11	
		Integrated	11.22±2.78	0.745
		Segmented	10.83±4.27	
Contralateral breast	D_mean_ (Gy)	All	0.98±0.46	
		Integrated	1.01±0.48	0.418
		Segmented	0.90±0.43	
	V_5_	All	2%±1%	
		Integrated	2%±2%	0.299
		Segmented	1%±1%	
Healthy tissue	EI	All	0.30±0.04	
		Integrated	0.31±0.03	0.094
		Segmented	0.28±0.05	

Abbreviations: *Vx*: percent volume of critical structures receiving a dose of x Gy; *EI*: external volume index; other abbreviations as in Table 2.

### Clinical data

All patients finished treatment per protocol. At up to 6 months of follow-up, 36 (42%) patients in total were identified to have ≥ grade 2 RD. Of these, 24 (28%) had grade 2, 12 (14%) had grade 3 RD, and 35 developed moist desquamation. The median time to the onset of moist desquamation was 6 (4–7) weeks from start of RT, and mostly occurred within 1–2 weeks after completion of treatment. The sites of moist desquamation were most frequently occurred in anterior axillary fold (32/35), and secondly in chest wall (12/35). Nine patients had moist desquamation in both anterior axillary fold and chest wall. All the area of moist desquamation occurred where bolus was applied. None was observed at the sites where moist desquamation most frequently occurred with conventional technique, i.e., the junction of chest wall tangents and IMN/SC field. The difference in the occurrence of ≥grade 2 RD between integrated (22/55) and segmented plans (14/30) was statistically insignificant (*X*^2^=0.35, *p*=0.55). In addition to RD, 23 (27%) patients experienced grade 2 swallowing pain. Only 2 (2.3%) patients were found to have grade 2 RP. No other severe acute toxicities were observed.

## Discussion

The conventional PMRT technique generally includes two opposed tangential photon beams for chest wall, and separate anterior fields for regional nodes with mixed photon-electron beams. This technique has several disadvantages. First, the tissue between the chest wall and supraclavicular region +/− IMN may be under or overdosed, because of the junction or overlap between the tangents and anterior fields, therefore potentially increasing normal tissues toxicities or reducing tumor control probability. Although many ways have been reported to be able to address the junction issue between chest wall and supraclavicular region [11,12], it’s difficult to eliminate the overlap between chest wall and IMN with geometric matching method. Second, the use of mixed beams for regional nodes may be associated with inhomogeneous dose distribution. In addition, the maximum depth of supra/infraclavicular region varied with patients’ anatomy [13] and body mass index (BMI) [14]; routine use of mean depth did not optimally cover intended targets for every patient, and might also result in overdose to some normal tissues in a portion of patients.

Recently, optimized CT-based treatment planning has been explored and found to offer better coverage of supra/infraclavicular targets than the empirically derived dose prescription that are commonly used, in Liengsawangwong’ dosimetric study [14]. The other study carried out by Krueger et al. [15] showed that a nine-field IMRT technique applied in patients receiving PMRT improved the coverage of chest wall and regional nodes while maintaining similar doses to heart and ipsilateral lung as conventional techniques. To further prove the feasibilities of inverse IMRT plans for PMRT in both dosimetric and clinical aspects, we therefore initiated this study to develop a linac IMRT technique with chest wall and regional nodes as a whole PTV, and to treat post-mastectomy breast cancer patients using this technique.

### Dosimetric characteristics of linac IMRT PMRT

**Figure 3:**
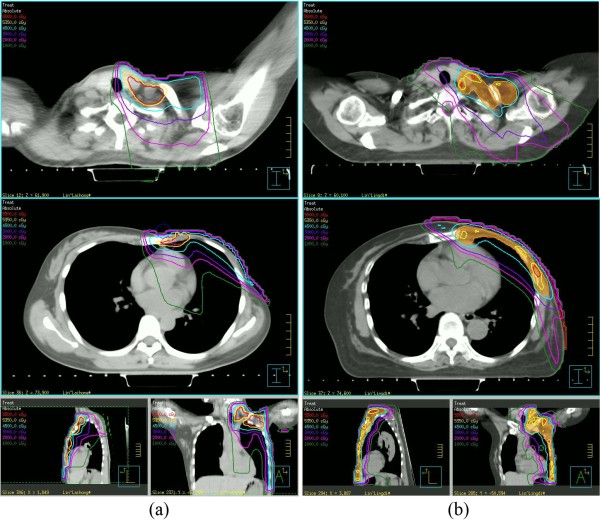
A direct comparison of dose distribution between IMRT (b) and conventional plans (a): red line:55Gy; yellow line: 53.5Gy; blue line: 45Gy; purple line: 30Gy; pink line: 20Gy; green line: 10Gy.

In dosimetric part of this study, the IMRT technique treating chest wall, supra/infraclavicular region, +/−IMN as a whole PTV was established by creating integrated or segmented plans for 85 patients after MRM. Both integrated and segmented plans were found to have adequate but similar PTV dose homogeneity and conformity. The percent volume of PTV receiving ≥110% of prescription dose was negligible and scattered in whole PTV. As an example, Figure 3 represents a direct comparison of dose distribution between IMRT and conventional plans. Hot spots and field junction issues associated with separate fields for regional nodes were basically eliminated.

Although conventional PMRT technique based on clinical target definition has been found to undertreat certain portion of target volume defined by RTOG or other contour guidelines [6], there has been no consensus whether new complex techniques to treat regional nodes and chest-wall as a whole PTV should become new standard replacing the traditional field design. As the target volume defined by CT-based contour are larger than that with clinical definition, undesired high dose to normal tissues is the major concern if dose constraints to normal tissue are not properly defined. As Fontanilla et al. [6] recently reported, when contour-based treatment plans were designed with traditional four fields without IMRT optimization, there exists an important increase in dose to the normal tissues. With a prescription dose of 50Gy, the mean ipsilateral lung V20 was 32% and 45% in left-sided and right-sided patients respectively [6]. This is slightly different to V20 reported at 28% in the current study. Rudat et al. [5] also found that tangential beam IMRT for irradiation of the chest wall of postmastectomy breast cancer patients could significantly decrease the dose-volume of the ipsilateral lung, and in patients with left-sided cancer the dose-volume of the heart compared to tangential beam 3D-CRT of the chest wall with the same target definitions, however, dose to the regional nodes was not evaluated. Treatment of the regional nodes and especially the IMNs increase the complexity of RT planning and delivery, and with certain techniques, may increase the risks of cardiac and pulmonary toxicity [16]. Thus, it is crucial to establish optimal IMRT techniques minimizing exposure to normal tissues and maintaining reasonable target coverage, and at least have well-defined dose constraints to normal tissue with prioritization in certain normal tissue when target volume coverage cannot be fully met. In the current study, both integrated and segmented IMRT plans showed reasonable normal tissue dose, while segmented IMRT plans conferred lower dose to nearby structures, such as contralateral lung, ipsilateral humeral head, and spinal cord, than integrated plans. This is largely attributed to the difference in beam selection for chest wall (mostly tangents) and for supra/infraclavicular nodes (with posterior-oblique ones). Hence, segmented IMRT plans had potential to better spare the surrounding normal tissues even if the number of total beams increased slightly (median 10 instead of median 7 in integrated plans).

### Toxicity profile following linac IMRT PMRT

Compared to the number of reports regarding IMRT techniques in PMRT [15,17-20], the toxicity profile is unclear in post-mastectomy breast cancer patients treated with IMRT technique, and available data mainly comes from patients treated with conventional techniques. In clinical part of this study, most patients tolerated treatment well. As with previous reports, RD was found to be the most common acute side effects following PMRT. The frequency of ≥grade 2 RD was 42% for this group of patients treated with IMRT technique, as compared to 40-90% for patients treated with conventional technique reported previously [21]. The occurring time to most serious RD and sites of moist desquamation were consistent with previous data as well. Although dosimetric analyses demonstrated that segmented plans were superior to integrated plans in the sparing of some nearby structures, especially the low dose volume of heart, ipsilateral lung and contralateral lung, we did not observe any difference in the proportion of patients experiencing RD between plans. Generally, the occurrence of RD is also associated with patient and treatment related factors, in addition to dose, such as beam energy, chemotherapy, application of bolus, etc. [22].

RP is another common side effect following PMRT. The frequency of symptomatic RP was reportedly 1-7% after local and regional nodes were treated to a total dose of 45-50Gy with conventional techniques [23-25]. In the current study, only 2 (2%) patients developed grade 2 RP, indicating that IMRT technique did not increase the risk of RP in breast cancer patients. According to reports in literature, a diversity of factors including age, BMI, dose-volume of normal lung, and chemotherapy contributed to the incidence of RP after PMRT [26]. Of these, the dose-volume metrics are major predictors of the risk of RP. Reducing the dose-volume of lung as much as possible remains to be one of the most important objectives of planning optimization.

### Limitations and future directions

As it has been highlighted earlier, there is no consensus whether RTOG contour guideline with contour-based treatment planning should be applied to each patient receiving PMRT. Studies using classic field definitions have yielded excellent local control [2-4], even if some part of the volume may have received lower dose than expected. Therefore, individual risk assessment and strict normal tissue constraints should be applied when treatment plan is to be generated based on today’s contour guidelines. There are certain limitations in this preliminary study. First, we did not assess the impact of IMRT technique on patients’ quality of life. Second, the association between RP and IMRT plans was not analyzed due to limited number of patients experiencing RP. Last, the follow-up period is not long enough to observe radiation-induced late toxicity, and biological effects of low dose irradiation remains a concern for patients treated with IMRT technique. Also, we did not perform dosimetric comparison with standard technique as the later one is based on clinical defined fields, with target coverage quite different with the current contoured definition. Further studies are therefore needed in near future to overcome these limitations. In addition, a randomized clinical trial comparing 3D conformal technique vs. IMRT for PMRT might help to answer some questions about patients’ tolerance, quality of life, and local control, and finally to determine a better PMRT technique.

## Conclusions

In this study, dosimetric analyses demonstrate that it’s feasible to design IMRT plans treating the chest wall and regional nodes as a whole PTV, in terms of both PTV coverage and normal tissues sparing. The post-mastectomy treatment with multi-beam IMRT technique was well tolerated by most breast cancer patients. Long-term follow-up with larger number of patients are needed prior to wide implementation of this technique.

## Abbreviations

BCS: Breast conserving surgery; MRM: Modified radical mastectomy; PMRT: Post-mastectomy radiation therapy; CT: Computed tomography; IMRT: Inverse-planned intensity-modulated radiation therapy; IMN: Internal mammary nodes; CTV: Clinical target volumes; PTV: Planning target volume; OAR: organ at risk; DVH: Dose-volume histograms; HI: Dose homogeneity index; CI: Conformity index; EI: External volume index; RD: Radiation dermatitis; RP: Radiation pneumonitis.

## Competing interests

The authors declare that they have no competing interests.

## Authors’ contribution

JM and JL participated in the study design, carried out the dose calculation, performed the statistical analysis, and drafted the manuscript. They contributed equally and shared the first authorship. JC conceived of the study, participated in its design, and helped to revise the manuscript. JX carried out the planning design. JC, CZ, and GC helped to collect data. ZZ and XG helped to revise the manuscript. All authors read and approved the final manuscript.
